# Felt and expressed needs regarding occupational health services from the perspective of micro-, small and medium-sized enterprises: a scoping review

**DOI:** 10.1186/s12889-026-28795-y

**Published:** 2026-08-05

**Authors:** Svea Suraj, Martin Ansgar Horn, Juliane Holzgräwe-Eichmann, Hanno Hoven, Stefanie Mache, Alexandra Marita Preisser, Marcial Velasco Garrido, Volker Harth, Robert Herold

**Affiliations:** Institute for Occupational and Maritime Medicine (ZfAM), University Medical Center Hamburg-Eppendorf (UKE), Hamburg, Germany

**Keywords:** Occupational health services, OHS, Needs, Micro-, small- and medium-sized enterprises, MSMEs

## Abstract

**Background:**

In the context of the changing need for health care and prevention due to climate, digital and demographic change, occupational health services are of enormous relevance, as many people can be reached. Of particular interest are the occupational health services in micro-, small- and medium-sized enterprises (MSMEs), as smaller companies are experiencing an unequal distribution of resources. To optimise the implementation in MSMEs, the aim of this scoping review is to present an overview of the MSMEs’ needs regarding occupational health services.

**Methods:**

The literature search was conducted through Embase, Pub Med, Scopus, and Web of Science. Additionally, a manual search was performed through the references of included sources. A protocol was registered with Open Science Framework. The screening was conducted by two reviewers. Eligible were peer-reviewed articles in English and German, published between 2000 and 2024 on the perspective of MSMEs regarding occupational health services. The articles were narratively synthetised. A critical appraisal was carried out.

**Results:**

A total of 1,252 sources were found during the search on August 26, 2024. Fifteen were included from the database search and two from the manual search. The studies used qualitative (*n* = 11), quantitative (*n* = 3) or mixed-methods (*n* = 3) approaches. The narrative synthesis identified five categories of felt and expressed needs with fourteen subcategories. MSMEs had a need for *collaborative culture* including communication, participation, trusting relationships and external support. The need for *knowledge and competencies* contained information, training and accessibility. *Organizational structures* subsumed the need for time, financial, technical resources as well as management commitment and simple solutions. Tailored legislation and less bureaucracy were categorised as needs for *political and regulatory context*. In the included studies, *a selection of specific interventions* such as flu vaccination, ergonomic solutions and psychosocial risk assessment were discussed.

**Conclusions:**

To optimise the implementation of occupational health services, this overview shows that political stakeholders and occupational health professionals should improve the organizational structures, political and regulatory context, collaborative culture with and the knowledge and competencies of MSMEs. Further research is recommended with the explicit aim of identifying the needs of MSMEs in each sector on occupational health services, psychosocial risk and assessments.

**Supplementary Information:**

The online version contains supplementary material available at 10.1186/s12889-026-28795-y.

## Background

Current changes in the work environment have a major impact on working conditions and therefore on employees’ health. The World Health Organization (WHO), International Labor Organization (ILO), European Agency for Safety and Health at Work (EU-OSHA) and the National Institute for Occupational Safety and Health (NIOSH) highlight the impact of demographic change, climate change, digitalisation and globalisation on the working conditions [1–7].

With new technologies like artificial intelligence and robotics, digitalisation are creating more flexible working conditions and growth opportunities for the working environment [8]. However, combined with globalisation, the labour environment is an unpredictable, fast-changing place for employees. A plethora of information, a lack of knowledge of how to use it, constant attention, fast-paced physical work, and flexible reaction to changes can cause physical and mental occupational hazards or illness [9, 10].

Amongst others the ILO highlights, that due to demographic change, older employees make up an increasing proportion of the workforce and are likely to feel more impacted by digital change, while fewer younger employees are available on the job market [1, 6, 11]. This shortage of skilled labour leads to more responsibilities for fewer employees and thus to potential stress or illness [12]. Additionally, the EU-OSHA states in its position paper that female workers and migrants will play an increasingly important role in a productive and healthy future labour market [6].

Furthermore, climate change introduces new health risks, particularly for outdoor workers and those in green jobs. EU-OSHA notes that new and intensified occupational hazards require a reassessment of risk levels in certain occupations [3]. Additionally, the ILO emphasizes that global warming will make heat stress, dehydration and extreme weather events increasingly common for all workers [2].

These challenges display the impact of changing work environments on the physical and mental health of employees. It is a very serious problem, considering the percentage of employed people (60%), according to the ILO, and the time they spend working every day [13].

These challenges lead to an increased need for healthcare and prevention in the workplace [9]. To master this, occupational health services (OHS) are of enormous relevance for prevention in the workplace and fundamental activities for prevention of work-related diseases or accidents [14]. According to the definition of the ILO convention No. 161 ‘occupational health services means services entrusted with essentially preventive functions and responsible for advising the employer, the workers and their representatives in the undertaking on the requirements for establishing and maintaining a safe and healthy working environment which will facilitate optimal physical and mental health in relation to work and the adaptation of work to the capabilities of workers in the light of their state of physical and mental health’ [15]. In the following, occupational health services are therefore understood in a broad sense, encompassing occupational health, safety, and prevention measures in relation to physical and mental health. These services can be classified in three categories. Interventions for primary prevention include e.g. ergonomic and risk prevention measures. Secondary prevention interventions include for example, employment screening, risk assessment, special and general health examination, while tertiary prevention services involve the treatment of occupational health injuries and diseases or return-to-work measures [14, 16]. According to the definition of the ILO convention No. 161 occupational health professionals are multidisciplinary such as occupational health physicians, nurses, safety specialist or psychologists are competent persons who provide either individual services related to workers’ behaviour or organisational services related to the environmental situation of the workplace [15, 17]. The ILO Conventions No. 155 [18] and 187 [19] provide international standards for national policies that guide the development and implementation of occupational safety and health. The WHO’s Global Strategy on Occupational Health for All [20] and Global Plan of Action on Workers’ Health [21] establish comprehensive frameworks for integrating occupational health into public health systems and prioritizing prevention to address the challenges posed by rapidly evolving working conditions.

Due to the large percentage of employed people (70% globally) in micro-, small- and medium-sized enterprises (MSMEs) [22] and considering ILO reports indicating that MSMEs are already affected by climate change and face increasing health risk for their employees [23], the occupational health service provision in these enterprises is of great interest. But the status quo is reported to be unequal between MSMEs and large companies [24]. According to the European Commission’s recommendation, companies are divided by number of employees, turnover and balance sheet total [25]. Only the employment definition is used here, as this category is the main data source in Eurostat’s Structural Business Statistics (SBS) database. While micro enterprises have one to nine employees, small enterprises have ten to 49 employees, medium-sized enterprises have 50 to 250 employees and large enterprises are those with more than 250 employees [25]. Even between MSMEs there are differences in provision of occupational physicians, safety experts and conduction of services. Micro enterprises are confronted with minimal provision of these services [24].

To optimise the implementation in MSMEs, specific needs of these differently sized enterprises must be satisfied and thus identified [26]. Throughout this paper, the term “needs” will be distinguished as “felt needs” and “expressed needs” according to Bradshaw’s classification [27]. While felt needs are associated with individual perceptions of want, expressed needs are felt needs that are translated into action and linked to demands [27]. Different analytic levels enable the research of the point of view either of individuals (micro level), organizations (meso level) or the society (macro level) [28, 29]. This scoping review is focused on the meso level, as the needs of enterprises will be analysed.

Existing reviews aim to present occupational health and safety activities in small enterprises [30], the implementation of legal requirements [31], indications of the effectiveness [32] or performance indicators in small companies [33]. Therefore, this study aims to summarise available scientific literature on the felt and expressed needs of occupational health services in micro, small and medium-sized enterprises.

In line with the Population, Concept, Context (PCC) framework [34], the research question is as follows: What are the needs of micro-, small- and medium-sized enterprises regarding occupational health services? The *Population* are the micro-, small- and medium-sized enterprises; the *Concept* is the felt and expressed needs for occupational health services in the defined broader sense and the *Context* are all sectors and all geographical regions of the enterprises.

## Methods

### Rationale

A scoping review was conducted due to the broad scope of the research question and the aim to map the available information on the needs of MSMEs regarding occupational health services to provide an overview and inform practical and scientific experts. According to the Joanna Briggs Institute (JBI) methodology, the scoping review is therefore the appropriate methodical approach for this study aim and scope [34]. The following information on the review methods are in accordance with JBI’s Preferred Reporting Items for Systematic reviews and Meta-Analyses extension for Scoping Reviews (PRISMA-ScR) checklist [35] (Additional file 1).

### Protocol registration

The prospective protocol for this scoping review was developed as recommended by the JBI manual chapter for scoping reviews and was registered with Open Science Framework. It contains detailed information on all stages of the review process including search strategy, screening, data extraction and data synthesis. The registration number is 10.17605/OSF.IO/HVGKA .

### Eligibility criteria

The inclusion and exclusion criteria for the study selection were derived from the research question and defined according to the PCC framework [34]. A summary table of these criteria is presented in additional file 2. The included population groups were micro-, small- and medium-sized enterprises (1 to 249 employees) in all sectors and all geographical regions. Eligible were articles on concepts like the needs of MSMEs regarding occupational health services with activities in primary, secondary, and tertiary prevention as well as those with a focus on behaviour or the working environment. Required topics for eligible articles included factors influencing injuries, exposures like risks and hazards, employees’ health status, experience and satisfaction, challenges and objectives of occupational health and the effectiveness of specific occupational health interventions.

Due to the expertise of the research team, the bibliographical fields considered were English and German. Only articles published since 2000 were included due to topicality. The accessibility of the abstracts and full texts was another eligible bibliographic field. Only peer-reviewed articles were assessed. Finally, primary studies such as qualitative, quantitative, mixed-methods studies and case studies were considered for the type of publication.

Studies which represented the perspective of occupational health professionals and other stakeholders were excluded. The following topics were not of interest for this scoping review as they covered separate research areas: papers dealing with specific occupational diseases, injuries and accidents, workplace health promotion, support for staff development, telemedicine or digital applications in occupational medicine, general healthcare or primary medicine and medical care unrelated to the workplace as well as studies on the COVID-19 pandemic. Studies on companies with more than 250 employees were also excluded. For the purposes of this review, editorials, letters, conference papers, dissertation summaries, commentaries, reflections, books and policy statements were not assessed.

### Information sources

To identify relevant information on the needs of MSMEs regarding occupational health services, the electronic databases Embase, PubMed, Scopus and Web of Science were searched based on their fit for the health topic and their complementarity to each other. The original interfaces were used based on existing access. This scoping review reflects the status of published articles at the time of the search. Peer-reviewed articles that were indexed in the databases were included in this scoping review to ensure a high standard of methodological quality and reliability in the evidence presented. The search was supplemented by a screening of the reference lists of included studies as a manual search.

### Search

The first author developed and executed the following search strategy in consultation with the co-authors. The Peer Review of Electronic Search Strategies (PRESS) checklist was used by the first author to review the search strategy. For replication, the search strategy for PubMed is described in detail in the following, while the adaption of the search string for the other databases can be seen in additional file 3. To search PubMed for relevant information on the needs of MSMEs regarding occupational health services in the defined broader sense, a search string was developed with the main topics of the subject according to the PCC scheme and the research question. English keywords were compiled for the topic blocks “occupational health”, “size of enterprises”, “enterprises” and “needs”. “Occupational health” was chosen, as it encompassed “occupational health services”.

The search strategy included the search fields “title and abstract” [Title/Abstract] to find the relevant information for the screening process. In addition, certain words of the search string were truncated (*) to find different word endings and were in inverted commas (“”) for phrase searches when two words are to be searched together rather than each word separately. The search string was developed using Boolean operators. The words within a topic block were connected with “OR” while different topic blocks were connected with “AND” to combine the search only with sources that contain all words in the title or abstract.

The search string for PubMed was adapted to the other databases using the same words in the topic blocks, search fields, truncation and phrase searches (Additional file 3).

### Selection of sources of evidence

The search results were exported into EndNote 20 (Clarivate Analytics, Boston, USA). After the duplicate’s removal in the first phase title and abstracts were screened according to the inclusion and exclusion criteria. If the title or abstract of a source met the exclusion criteria, the entry was directly excluded. In the second phase, all sources with full text were assessed. To find the full texts on the articles, data bases were searched, and the institution’s librarian was asked for exclusive access. If the title and abstract of a source met the inclusion criteria, but the full text didn’t, the source was excluded. To minimise selection bias, only essential bibliographic fields like title, abstract, and full text were visible during the screening process, while all other fields were blinded. Blinding reviewers to non-essential bibliographic information reduce the influence of other factors and to ensure that study selection is based solely on content relevance and quality [34, 35]. To increase consistency, two independent researchers (SvSu & MAH) checked the sources in title, abstract and full text screening and documented their decisions in Rayyan (Rayyan Systems Inc., Ottawa, Canada). After the first 10% of sources, the screeners compared the results and discussed the procedure and compliance of the inclusion and exclusion criteria. If there were discrepancies regarding a source, a third researcher (JH-E) was consulted. This procedure was justified by the recommendations of the JBI manual [36].

### Quality assessment

The process of assessing the research evidence is mandatory for systematic reviews, but not intended in the original framework of a scoping review from Arksey & O’Malley [37]. However, there are recent recommendations that a critical appraisal should also be carried out in scoping reviews in order to assess the quality of the included studies [36, 38]. Brien et al. stated that a critical appraisal of the studies will lead to a better interpretation of the results of a scoping review [39]. To assess the methodological quality of the included sources and determine possible bias in design, conduct and analyses, standardized critical appraisal tools from the JBI were used [40]. These tools were approved by the JBI Scientific Committee after a comprehensive peer review [41]. The checklists for qualitative research and analytical cross-sectional studies contain 8 to 10 methodological criteria for examining sources [40]. The appraiser could assess whether the criterion was met or not, was unclear or not applicable. Due to resource constraints, only one author (SvSu) assessed the included sources and discussed the results with the co-authors. The percentage of criteria met in relation to all criteria was reported.

### Data extraction

Entities for the data extraction were qualitative data fragments (topic, aim and main findings related to the objective of this scoping review), descriptions of the used methods (study designs, sample sizes, population characteristics) and source metadata (authors, language, country and year of publication). The aim of providing an overview of the needs of micro-, small- and medium-sized enterprises regarding occupational health services justified the extraction of the qualitative data fragments. To contextualize the qualitative data, descriptions of the used methods and metadata of the sources were also extracted.

These entities were discussed with the research team, and a data extraction form was developed. Using this form, data was extracted from three included sources in a training phase, before data was charted from 10% of all included sources in the second phase. After a discussion with the research team about the procedure, no changes were made to the form, and the final extraction took place in the last phase. All data from the included papers were charted by one reviewer (SvSu). The three extraction stages in this scoping review served to practice and optimise the extraction progress. The data extraction results were discussed with the research team.

### Synthesis of results and data analysis

Following the suggestions from the JBI manual, the main findings related to the aim of this scoping review were categorised using narrative synthesis [34]. The results are presented narratively in the categories explained below. Furthermore, source metadata and description of the methods used were analysed and summarised descriptively by frequency and relation to define the context of the qualitative findings.

## Results

### Selection of sources of evidence

**Fig. 1:**
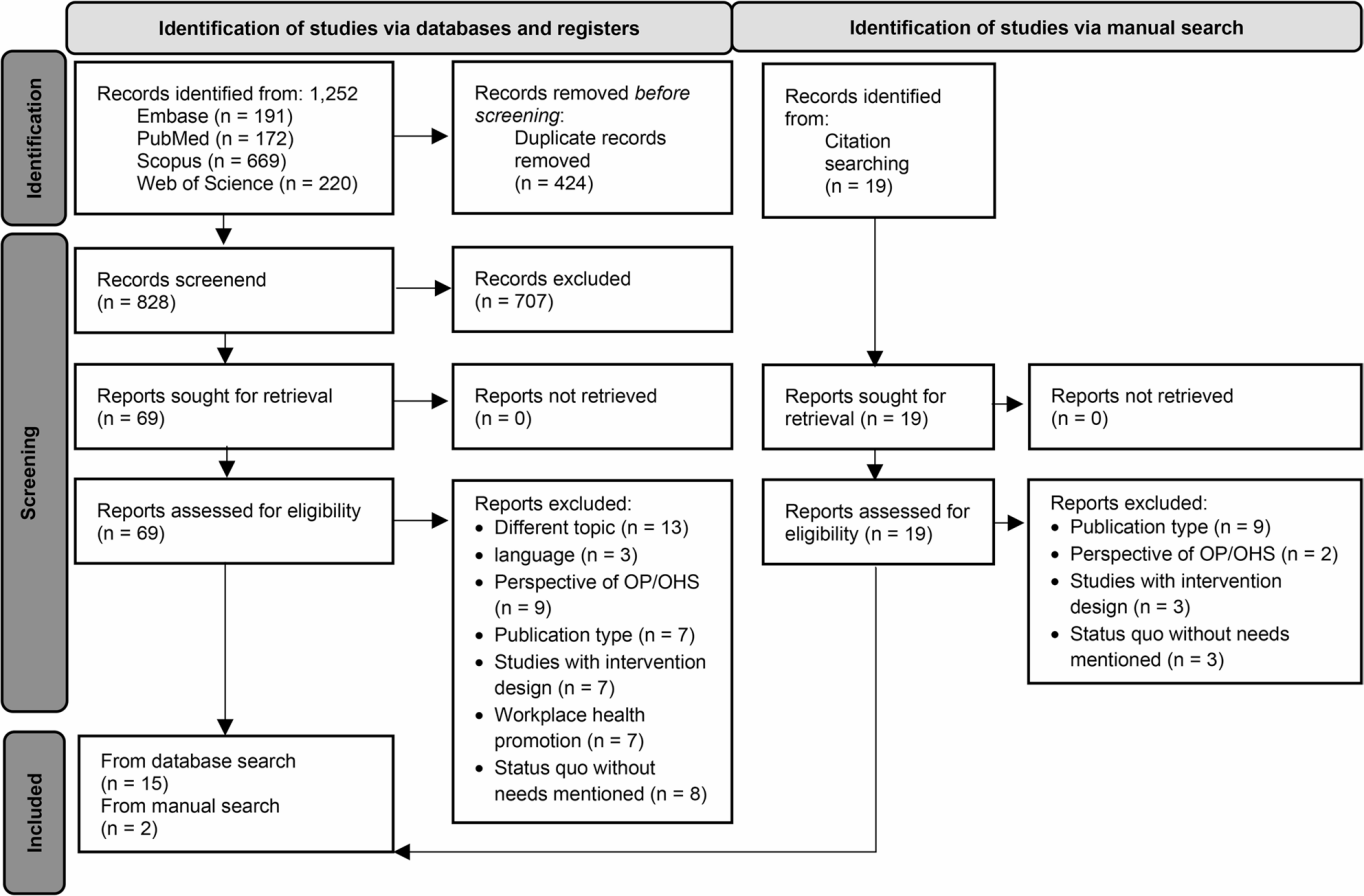
PRISMA-ScR flow diagram of the study selection process

On August 26, 2024, the search returned 1,252 records in four databases. Prior to the screening process, 424 duplicates were removed. Two reviewers (SvSu & MAH) screened 828 records according to the eligibility criteria in the title and abstract. The study selection process is presented in a flow diagram (Fig. 1). The consistency between the reviewers in the title and abstract screening was very good (Cohen’s kappa κ = 0,84). Out of these 828 records 69 were sought for retrieval and assessed for eligibility in the full text. No studies were excluded due to lack of full-text availability, as the full texts for all included articles were accessible. According to the eligibility criteria, 15 papers were finally included for data extraction and analysis. The two reviewers achieved a good level of agreement in the full text screening (Cohen’s kappa κ = 0,61). Reasons for exclusion were topics not relevant (*n* = 13), a language other than German or English (*n* = 3), the perspective of occupational health professionals and not the perspective of the MSMEs (*n* = 9) or the publication type (*n* = 7). Papers describing the status quo without mentioning the needs of MSMEs (*n* = 8) or workplace health promotion (*n* = 7) were excluded. Further, studies with intervention design (*n* = 7) weren’t considered for this scoping review as it was intended to summarise the daily needs of the MSMEs, not their opinion about certain programmes or projects being tested with intervention and reference groups. To enhance transparency, a list of excluded studies has been provided in additional file 4.

In addition, a manual search was carried out to identify relevant entries in the literature cited from the 15 included sources. Here, 19 records were searched and assessed according to the eligibility criteria only by one reviewer (SvSu). Two records were added, resulting in 17 studies being included in this scoping review. Reasons for exclusion were studies with intervention designs (*n* = 3), publication type (*n* = 9), the perspective of the occupational health professionals (*n* = 2) or description about the status quo without mentioning the needs of MSMEs (*n* = 3).

Most of the studies were conducted in Europe in the last 10 years, 15 were published in English and two in German (Table 1). Consistent with the broader definition of occupational health services and the inclusion of interrelated concepts, the majority of records addressed occupational health and safety, while others focused on psychosocial risks, well-being, and prevention. More than 60% of the studies had a qualitative design with face-to-face interviews, telephone interviews or focus groups. The others were either cross-sectional surveys or mixed-method studies. While the MSMEs’ perspectives were stated in all papers, 30% of them followed a multi-perspective approach. However, the results of these cases were divided, so that only the MSMEs’ perspectives were synthesized. The target group of 42% of the included studies were owner-managers and 18% were safety officers in the enterprises. In two studies, the target group was not specified. More than half of the enterprises that participated in the included studies belonged to the secondary sector, which included the processing of materials and industrial production such as garments and mental products or the manufacture of furniture [42]. While 30% were in the tertiary sector with focus on services like cleaning and car repair, only 14% came from the primary sector, which relates to the extraction and production of raw materials [42].

**Table 1:** Overview of the included studies (*n* = 17)

Author, Year	Country of Conduct	Study Design	Participants	Key findings on needs
Amler et al. (2019) [24]	Germany	Mix-methods: survey study online and qualitative telephone interviews	724 owners of companies of all sizes (micro, small, medium)	Owners feel a lack of information about occupational health and the regulations in every enterprise size, but information needs differ between the sizes.
Barbeau et al. (2004) [48]	USA	Qualitative interview (not specified)	22 worksites persons most responsible of health and safety	There is a need for more employee participation in small enterprises. Improvement in management commitment, training and education is needed. Ergonomic issues should be a priority for occupational health effort.
Benning et al. (2022) [46]	Netherlands	Mix-methods: survey study online and qualitative interviews	79 SME representatives (e.g., owners, HR professionals and occupational health and safety officers) & 18 enterprise representatives	Small enterprises need available resources (both finances and staff), complexity of implementation of measures, awareness knowledge and expertise, employer and worker commitment, workers’ openness for measures, communication, workers’ trust and autonomy and integration in organizational policy.
Cagno et al. (2016) [53]	Italy	Qualitative telephone interviews	58 owner-managers and the safety officers of small and medium-sized enterprises (SMEs)	The most characteristic drivers for occupational health are external support of consultants, the availability of knowledge of effective interventions and collaborations with networks of companies.
Champoux and Brun (2003) [49]	Canada	Qualitative telephone interviews	223 owner-managers of small enterprises	Small enterprises have a need for information about programmes, easy access to possible solutions, employee participation in safety management, technical expertise or financial help.
Coppens et al. (2023) [47]	Albania, Australia, Finland, Germany, Hungary, Ireland, Kosovo, the Netherlands and Spain	Survey	65 (1) academics with expertise in workplace mental health promotion and occupational health; (2) steering group members or directors of SME organisations; (3) steering committee members and representatives; and (4) occupational health specialists and representatives of associations, labour unions and advocacy groups	There is a need for communication and information about psychosocial interventions in small enterprises as well as financial help, best practice examples and time management.
Garnica and Barriga (2018) [54]	Brazil	Survey	38 owners, 15 auditors and 3 consultants	The main barriers to the implementation of occupational health and safety management systems are that managers tend to blame employees and government for difficulty in implementing.
Holt and Powell (2015) [50]	United Kingdom	Qualitative telephone interviews	91 SME owners/managers	Small enterprises across Greater Manchester need appropriate information and support against sickness absence and sickness presenteeism.
Kvorning et al. (2015) [59]	Denmark	Qualitative telephone and face-to-face interviews	20 SME owners/managers	Small enterprises need access to economic/financial support, networks, trusting relationships and technical equipment to influence the motivation of owner-managers.
Masi and Cagno (2015) [45]	Italy	Qualitative telephone interviews	58 safety officers of Small and Medium-sized Enterprises (SMEs)	Barriers to occupational health are bureaucracy, economic resources and a lack of information.
Masi et al. (2014) [51]	Italy	Qualitative face-to-face interviews	5 SME safety officers	Small enterprise employers and workers need to be participating in the design and the implementation of occupational health. There must be an effective mediator between them and the government.
Micheli and Cagno (2008) [58]	Italy	Mixed-Methods: preliminary qualitative interviews and then a survey	109 SME	Small enterprises need more incentives to sustain provision of training in safety activities, practices examples and tailored legislative.
Micheli et al. (2018) [52]	Italy	Qualitative interviews (not specified)	40 owner-managers or OSH managers of SME	Small enterprises have a need to inform the personnel about occupational health importance and participation, less bureaucratic requirements and communication between stakeholders.
Nowrouzi et al. (2016) [55]	Canada	Survey	153 workers, supervisors/managers/business owners	Small enterprises need tailored legislation, support in conducting external safety inspections. While workers need training in recognizing usage conditions and reporting them.
Pavlista et al. (2021) [56]	Germany	Qualitative interviews (not specified)	18 owners and managers	Small enterprises need easy access to psychosocial risk assessment material, external support form experts, simply solutions and information campaigns.
Stynen et al. (2023) [44]	Netherlands	Qualitative interviews and focus group	15 higher managers in SME	Small enterprises need trust and confidentiality, a well classified role of OP and more information and work-related absence/illness.
Zimber et al. (2024) [57]	Germany	Qualitative interviews (not specified)	27 managers of SME	Small enterprises need more resources (time and financial), information on occupational health services and social support.

### Critical appraisal within sources of evidence

To improve the quality of this scoping review and the interpretation of the results of the included sources, a critical appraisal was conducted using the tools of the JBI [34, 40, 43]. The checklist for qualitative research was used for 14 papers and the checklist for analytical cross-sectional studies for 6 included studies. The results are presented in the additional file 5. In three cases, both checklists were used, as these were mixed-method studies. Overall, the qualitative studies met 70 to 90% of the 10 criteria for qualitative research. Most of the unmet criteria were the lack of statements that culturally or theoretically located the researcher and addressed the researcher’s influence. Although these statements would enhance the quality of these studies, the results were still used in this scoping review. Of the 6 cross-sectional studies, the criteria met ranged from 63 to 100% of the 8 categories. Almost all these studies lacked information about confounding factors and strategies to deal with them. Even though these studies would have benefited from reporting this information, the results of all cross-sectional studies were also used in this scoping review.

### Synthesis of results

The narrative synthesis of the key findings from the 17 studies identified various subcategories of felt and expressed needs, reflecting the perspectives of micro, small, and medium-sized enterprises. These 14 subcategories were narratively grouped into five overarching main categories, which are presented in the overview table (Table 2). These main categories represent a higher level of abstraction, three of them include both felt and expressed needs. Therefore, when referring to these higher-level categories, the term ‘needs’ is used in a general sense.

**Table 2:** MSME’s felt and expressed needs regarding occupational health services in the broader scope

Main categories of needs	Subcategories of needs	Results of the included studies
Collaborative Culture	Felt need: Communication	• Effective, clear and transparent [44–46] • Right channels [45] • Specific communication strategies to address MSMEs’ preferences [46] • About mental health [47]
	Felt need: Participation	• Of employee [48–52] • In design and implementation phase [51]
	Felt need: Relationships	• External and internal stakeholders [46, 53–56] • Trust [46] • Confidentiality [44] • Important regarding mental health [47]
	Expressed need: External support	• Role clarified to all stakeholders [44] • Mediator between MSMEs and the government [51]
Knowledge and Competencies	Felt need: Information	• On occupational health care, mental health and risk assessment [24, 44, 47, 49, 51, 53, 54, 57] • Quality and availability important [51, 53] • Information campaigns [56]
	Felt need: Access	• Accessible solutions, best practices and material for (psychosocial) risk assessment [49, 50, 53, 56, 58] • Shared platforms [51]
	Expressed need: Training	• About safety behaviour and psychosocial risks for employees [48, 49, 52, 55]
Organizational Structures	Felt need: Management commitment	• In general, and specifically to psychological risk prevention [46–48]
	Expressed need: Time	• Particularly in the implementation and evaluation phase of an occupational health intervention [44–48, 57]
	Expressed need: Financial	• Low cost, free services, economic incentives or financial help and support [46–48, 50, 55, 57]
	Expressed need: Technical	• Support and training [49, 50]
	Expressed need: Practical solutions	• Practical and simple interventions [56, 58]
Political and Regulatory Context	Expressed need: Tailored legislation	• Customised for the daily business [45, 51, 54, 55, 58]
	Expressed need: Less bureaucracy	• Reduce bureaucratic requirements [45, 51, 52, 54]
Selection of Specific Interventions	Expressed need:	• Flu vaccination [50]
		• Psychosocial risk assessment [47, 56]
		• Ergonomic solutions [48]

### Collaborative culture

According to the 17 studies included a need for collaborative culture such as communication [44–47, 52, 54], participation [46, 48, 49, 51, 52, 59], external support [53, 56] and trusted relationships [46, 59] played an extremely relevant role for MSMEs. The expressed need of MSMEs for external support regarding occupational health services in the broader scope was identified in various studies. It was important that this support came from experts, consultants or occupational professionals [44, 53, 56]. The qualitative study by Stynen et al. showed that the role of external support should be clarified to all stakeholders, as MSMEs had a critical perception of professionals [44]. Concerns should be taken seriously for the benefit of the preventive strategies in these enterprises [44]. In terms of content, the expressed need for external support against sickness absence, for prevention efforts and in conducting external safety inspections was highlighted [46, 50, 55].

Furthermore, Masi et al. pointed out that external support was needed as an effective mediator between MSMEs and the government [51]. In addition, relationships with associations and networks were an important felt need of MSMEs to promote occupational health [53, 59]. In the qualitative study by Kvorning et al., it was argued that MSMEs owners may become aware of occupational health and safety programmes through these types of relationships and that they may consequently be more inclined to develop internal motivation to introduce them [59].

Within the enterprises, a felt need for employee involvement and participation was mentioned [52]. Employees should be engaged in the design and implementation phase of occupational health and safety interventions [60]. According to the mixed-methods study by Benning et al., preventive efforts by external support were effective, with the employees being involved at every phase [46].

In general, trust and confidentiality in external and internal relationships were important felt needs of MSMEs regarding occupational health and safety programmes and preventive health measures [46, 59]. Similar to the inclination factor of owners developing internal motivation through relationships, a trusting relationship with either the labour inspector, the employer associations or other networks is seen as even more important according to Kvorning et al. [59]. Especially with a focus on mental health, confidentiality of professionals was highly valued in MSMEs emphasised Stynen et al. [44].

Moreover, communication was the key felt need when it comes to relationships with stakeholders. MSMEs needed effective, clear and transparent communication between employees and external stakeholders [44, 46]. In turn, there was an expressed need in the MSMEs that the external stakeholders need a specific communication strategy through the right channels [45]. Benning et al. found that governments, regulators, sector associations and other intermediaries need to develop specific communication strategies to address MSMEs’ preferences for purposeful preventive health measures [46].

Furthermore, Champoux & Brun analysed four different profiles of enterprises by the activities and management style. Although this study primarily addresses occupational health and safety management, the various enterprise profiles presented may also serve as a foundation for developing targeted communication strategies during the implementation and delivery of occupational health services [49]. The largest group with 52% were the enterprises in profile 2, which are described as ‘inactive, traditional and unstructured’. This was followed by the enterprises in profile 4 (23%), which were labelled as ‘active, participatory and structured with networks.’ Only 18% of the enterprises fell under the category ‘active, participatory but unstructured’ (profile 3), whilst 7% could be characterised as ‘inactive and uninformed’ in profile 1 [49]. The study did not explain in detail how the communication should take place in each group.

In the multi-perspective study by Coppens et al., the felt need for regular communication about mental health became clear [47]. Half the participants indicated the felt need for information, tools and advice on creating mentally healthy working conditions (58.5%), on work stress and burnout (53.8%), on establishing policies to create mentally healthy workplaces (50.8%) and on detecting and dealing with mental health problems by management and human resources staff (50.8%) [47]. These topics should be addressed in the context of trusting relationships with professionals and treated confidentially [44].

### Knowledge and competencies

In addition to collaborative culture, knowledge and competencies in terms of information, access and training on occupational health services in the broader scope was also an important category of the MSMEs’ felt and expressed needs. In all papers, the felt need for information and knowledge about occupational health and safety [49, 59], risk [24, 46, 56] and preventive measures [44, 46], work-related illness [44], health conditions at work [47, 55, 57, 59], sickness presenteeism [50], psychosocial risks and regulations [47, 56] were the most common results. Specifically, the mixed-methods study by Amler et al. showed that MSMEs indicated a felt need for information on occupational health care (42%), mental health (33%) and risk assessment (30%) [24].

The quality and availability of this information were a relevant issue for MSMEs [46, 53]. Masi & Cagno mentioned that the ideal intervention process was based on the best available knowledge and information, while the actual process was experience-driven, which is why MSMEs stated a felt need for available information on best practices and solutions [60]. The qualitative results of Cagno et al. confirmed that the focus on the availability of knowledge about effective interventions was the second most perceived driver in their study [53]. According to Pavlista et al., there was a felt need for various information campaigns on these topics from professional associations, guild health insurances or other occupational health services providers [56].

Moreover, different studies pointed to the expressed need for accessible solutions, best practices and material for psychosocial risk assessment [49, 50, 53]. Masi & Cagno reported on the idea of improving existing databases or shared platforms and promoting them better among MSMEs [60]. In terms of general know-how, there was a felt need for owners and managers of MSME to improve their health literacy [56]. Employees, on the other hand mentioned the expressed need for training and education about safety behaviour and psychosocial risks [48, 55, 58]. The cross-sectional study by Nowrouzi et al. allowed for a deeper understanding of the topic, as the employees stated the expressed need to recognise unsafe conditions and training in how to report them [55].

### Organizational structures

An equally important category of the expressed and felt needs was summarised as organizational structures. All studies mentioned time [44–48, 57], financial [46, 47, 49, 55, 57, 59] and technical resources [49, 59], as well as management commitment [46–48] and practical solutions [56] as some sort of need for MSMEs. The time component was particularly significant in the implementation and regulation of occupational health and safety measures, as well as preventive strategies [44, 48, 57]. In their qualitative study, Masi & Cagno, for example, examined the expressed need for time particularly in the implementation and evaluation phase of an occupational health and safety intervention in MSMEs [45]. In addition, Zimber et al. mentioned in their qualitative study with 27 managers of MSMEs that they need time and attention to work through the process of occupational health and safety, which is difficult considering the simultaneous need to perform regular work tasks [57].

However, financial resources were issues as well. Low cost, free services, economic incentives or financial help and support were expressed needs of MSMEs to encounter these issues [50, 58]. Nowrouzi et al. showed in a cross-sectional study, for instance, that MSMEs lack the financial resources to hire a qualified occupational safety inspector [55].

In addition, the need for technical resources such as support and training were expressed [50]. Masi & Cagno identified that support was needed particularly in the design and implementation of occupational health and safety interventions [45].

Generally, programs and solutions should be practical and simple [56]. Micheli & Cagno emphasised the expressed need for examples of good practices and access to this information in a mixed-methods study [58]. Moreover, Kvorning et al. demonstrated that MSMEs expressed the need to align occupational health and safety programmes with their daily work routines [59]. The findings of Benning et al. showed that management commitment in general and specifically to psychological risk prevention was a felt need to ensure the realisation of preventive health measures [46].

### Political and regulatory context

Political and regulatory context made up the fourth category of synthesised needs. MSMEs had the expressed need for tailored legislation and less bureaucracy in terms of occupational health services in the broader scope [45, 57, 58]. It was difficult for them to implement the stated regulations on occupational health, as they were not customised for the daily business of these enterprises. MSMEs had to deal with regulatory paperwork on top of their normal business and felt overwhelmed by the multitude of regulations [24, 59].

In a survey study, Garnica & Barriga identified bureaucracy and strict legislation as the main barriers to the implementation of occupational health and safety management and consequently, to the implementation of occupational health services [54].

In addition, Masi & Cagno concluded that tailored legislation was necessary for these enterprises to implement these services or at least modify some aspects of regulations, for example paperwork and bureaucracy [60]. Moreover, Micheli et al. reported that the need for less bureaucratic requirements was especially expressed in the design and implementation phase, based on an interview study [52]. They further suggested that legislators should understand the needs of MSMEs and reduce bureaucratic requirements to improve the implementation of occupational health and safety interventions in these enterprises [52].

### A selection of specific interventions

The results of four studies presented a selection of specific interventions, which the MSEMs addressed during the conduction in exploratory designs about the status quo. Barbeau et al. found in their qualitative study that ergonomic issues should be a priority for new efforts in occupational health and safety programs [48]. Holt & Powell stated that the provision of preventative services in the workplace such as flu vaccines was needed, but the costs should be reduced for the MSMEs [50]. MSMEs would be very interested in having the vaccine provided by an occupational health professional on-site instead of employees having to leave their workplace independently to go to their general practitioner. Furthermore, Coppens et al. presented quantitative data focussing on psychosocial interventions for employers and employees [47]. While Pavlista et al. discussed in a qualitative study the implementation of psychosocial risk assessment in enterprises as an expressed need [56].

### Main categories of needs by country

To account for the impact of national contexts on occupational health, safety, and prevention, the results are presented by country. Across all European studies, MSMEs consistently reported a felt need for information related to occupational health, safety, and prevention. Overall, collaborative culture emerged as the dominant category of needs, with MSMEs in Europe mentioning needs for communication, trusted relationships, and external support [24, 44, 46, 50, 51, 53, 56–59]. Whereas knowledge and competencies in occupational health, safety, and prevention are the predominant category of needs in the studies conducted in non-European countries, with MSMEs in each study consistently emphasizing an expressed need for training in these areas [48, 49, 54, 55].

Country-specific analysis indicates that collaborative culture was the predominant main category of needs among MSMEs in the USA [48], Brazil [54], the Netherlands [44, 46], Italy [45, 52, 53, 58, 61], Denmark [59], and Germany [24, 56, 57]. In Canada, knowledge and competencies were most frequent main category of needs [49, 55], while organizational structure was the main category of needs in the UK [50] and in the multinational study [47].

## Discussion

In this scoping review the felt and expressed needs from the perspective of micro-, small- and medium-sized enterprises were summarised and mapped. The results can be used to optimise and adapt the implementation of occupational health services in the broader sense of occupational health, safety and prevention in these enterprises. Seventeen studies were examined in accordance with the PRISMA guidelines of the JBI [35]. The narrative synthesis resulted in five categories of needs with 14 subcategories. The felt and expressed needs in relation to communication, participation, external support and trusted relationships were summarised in the category of collaborative culture. The need for information, access and training regarding occupational health services in the broader scope can be subsumed into the category of knowledge and competencies. The category of organizational structures includes the MSMEs’ need for time, financial and technical resources, management commitment and practical solutions. The need for tailored legislation and less bureaucracy about occupational health services in the broader scope is categorised as political and regulatory context. The remaining category includes the needs for a selection of specific interventions particularly mentioned in the included studies such as flu vaccination, ergonomic improvements, psychosocial interventions and risk assessment.

In terms of the relationships between the categories, a pattern emerges. Needs for information, financial incentives, and external support are frequently mentioned across various included studies. Regardless of methodological approaches, regions, sectors, or participants, different studies consistently report that MSMEs require information on occupational health, safety, and prevention, as well as increased financial support for implementation and external assistance to manage all requirements simultaneously.

A political and regulatory context characterized by reduced bureaucracy and tailored legislation for occupational health services, particularly in Europe, could enhance the organizational structures of MSMEs. This may result in increased availability of time, technical resources, or financial incentives, thereby fostering greater management commitment to utilizing external support for occupational health services. External support could lead to more practical solutions in line with the daily business, which is of great value for easy implementation. Hasle & Limborg also concluded in their review that occupational health activities were more successful when the solutions were low cost, simple and delivered through personal contact [30]. However, in contradiction to this, simple solutions can be affected by strict government regulations. Similarly, external support and management commitment can diminish each other’s impact if motivation decreases on one side or if one party assumes the responsibilities of the other.

Nevertheless, improved organizational structures, trusting relationships with MSMEs leaders and external support could motivate employees and enable their participation in the implementation phase of occupational health services. Halonen et al. also discovered in their systematic review on characteristics of good collaboration between employers and occupational health service providers, that dialogue, shared goals, frequent contact and trust are key characteristics [62].

External support is also associated with communication about relevant information, access to occupational health services and training on various topics such as physical and mental health or risk and safety management. This information is essential for MSMEs to implement occupational health services. These results are consistent with the conclusion of Breslin et al., who found that training and safety audits improved MSMEs’ safety outcomes, although few studies in their systematic review adequately evaluated small business interventions [32].

In turn, communication between MSMEs especially in secondary sector and external stakeholders about access, practical solutions and the selection of specific services could bring about a change in tailored legislation on occupational health services. In general, the results that clear communication was a key felt need are in line with the conclusion from Tejamaya et al. that inter- and intra-organization communication are influencing the occupational health and safety management in MSMEs [63]. Schulte et al. added that different company sizes require different communication approaches [64].

According to the definition of Bradshaw [27], it can be pointed out that the felt needs of MSMEs regarding occupational health services in the broader scope represented in the included studies were relationships, communication, participation, information, access and management commitment. These interactions are perceptions of the MSMEs and can be associated with wanting those to happen [27]. The expressed needs included external support, training, practical solutions, financial, time and technical resources, tailored legislation, less bureaucracy and special interventions. These are associated with services, which the MSMEs demand to improve the occupational health service implementation [27]. These expressed and felt needs on the meso level of the enterprises must be seen in the context of the needs of the individual employees on the micro level as well as of the society on the macro level [28, 29].

Overall, these needs are addressed within the broader context of occupational health services. According to the definition provided by ILO Convention No. 161, occupational health services encompass preventive functions, the provision of a safe and healthy working environment, the promotion of physical and mental health, and the adaptation of work [15]. Due to this broad definition, occupational health services overlap with occupational safety and health, as well as with related interventions and management approaches. These concepts naturally interact, as occupational health and safety [65] constitutes the broad field within which occupational health services [15] are delivered through occupational health and safety interventions [66]. In enterprises, occupational health and safety management systems [67] are often in place to organize these interventions and the provision of services. In this study, these concepts are considered from a broader perspective, united by the overarching goal of implementing measures to improve employees’ mental and physical health and to ensure safe working conditions.

Furthermore, the felt and expressed needs must be placed in the context of the changing work environments, as well as legal, time and sectoral factors. Collaborative culture are of great interest as employees have different age groups, values and socialisations [68]. Stakeholders need to be aware of these differences in communication, participation and relationships [69]. The framework of technical resources should also be related to the age of the employees who are to participate in occupational health services [11]. Not only does digitalisation entail different mental and physical risk and needs of employees, but communication, access, training and information channel may also differ [69]. According to the EU-OSHA, the increasing presence of female workers and migrants is contributing to the evolving nature of workplaces [6]. This demographic shift, encompassing variations in age, gender, and origin, should be systematically considered when fostering a collaborative culture. Aspects such as participation, interpersonal relationships, and communication must be adapted to address more diverse workforces.

Furthermore, the presented needs should be viewed in a temporal context. More publications appeared in the last 10 years, which indicates an increasing interest in the topic with more included studies published between 2014 and 2019 and in 2023. Concerning the expressed need for technical resources, it must be pointed out that only publications prior to 2015 contain this information [45, 49, 50, 59]. Ten years later, it can be assumed that digitalisation in the working world has changed the expressed need for technical resources for enterprises today.

Most of the included studies were conducted in Europe, mainly in Italy, the Netherlands and Germany. But most needs are evenly represented across all countries included. The results should be considered in the legal context and different organisation of occupational health services in these countries. In particular, felt and expressed needs in the categories of organizational structures [32–37] and political and regulatory context [40–42, 47, 48] should be contemplated in the legal context of each country. These considerations are also closely linked to global occupational health policies established by the WHO and the ILO. In particular, the categories related to collaborative culture should be contextualized with reference to ILO Conventions No. 155 [18] and 187 [19] as well as WHO’s Global Strategy on Occupational Health for All [20] and Global Plan of Action on Workers’ Health [21]. These international frameworks significantly influence occupational health practices in MSMEs and shape the perception of occupational health needs within these enterprises.

In terms of content, it should be mentioned that this scoping review thematises the perspective of MSMEs. Nevertheless, the responses of owners, safety officers and other participants could differ, and hence the results may not have been comparable. The fact that most participants work in MSMEs in the secondary sector leads to an overrepresentation of this sector in this review. Globally most employees work in the tertiary sector according to the international labour organization [13]. Nevertheless, the needs identified in the 17 included studies are distributed evenly among the sectors.

Finally, some research gaps identified in the included studies are of interest. Methodologically, several studies suggest that longitudinal research [57] and the inclusion of workers’ perspectives [46] could enhance current knowledge. Others criticize the reliance on self-reports regarding the situation in MSMEs [44] and recommend that assessments by external inspecting institutions would provide a more objective view. In terms of content, further investigation is needed into the (cost-)effectiveness of implementation measures [47], as well as into the knowledge and potential knowledge gaps of MSME managers concerning the implementation of occupational health, safety, and prevention [24].

### Recommendations for political and occupational professionals

To improve the unequal provision of occupational health, safety and prevention in MSMEs, their needs should be met by both political stakeholders and occupational professionals. To optimise the political and regulatory context, political stakeholders especially within Europe should consider less bureaucracy and tailored legislation for MSMEs. It should include flexible time resources, customised for the daily business of these enterprises, technical support as well as financial support or incentives. In addition, trusting relationships should be built with communication strategies through the right channels. External support and information campaigns can help improve the knowledge and competencies of MSME managers regarding occupational health. Given the changing working environment and the resulting challenges for small enterprises and their employees’ health, this topic should be given high priority.

In addition, occupational health professionals and service providers should communicate clearly and effectively to establish trusting and confidential relationships with MSMEs, particularly in the secondary sector. The goal should be management commitment and thereby employee participation. Communication and information from external professionals on physical and mental health or safety management is crucial. These topics, practical solutions, access to information and services should be communicated through the right channels to improve the implementation, commitment and participation in every phase of the process. Occupational professionals should also consider the expressed needs of MSMEs for time, financial and technical support to customise the services for their daily business. The selection of specific interventions presented such as flu vaccination, ergonomic solutions or psychosocial risk assessment could be considered.

### Recommendations for further research

The limited number of sources on the needs of MSMEs indicate that this topic has not been well explored in recent studies. To close this research gap and optimise the situation regarding occupational health, safety and prevention of the MSMEs, further research should be conducted with the explicit aim of identifying their needs. Furthermore, after identifying the relevant needs, future research should assess the enabling conditions and factors that support the fulfilment of these needs.

An interesting topic of research would also be the technical resources regarding occupational health services of small enterprises, as only the included studies published 10 years ago showed a need for improvement. Due to digitalisation, it is unclear whether this need still exists today. Deeper understanding of the right communication channels and strategies of different stakeholders are also of great interest, as the need was stated but not explained in detail. As for the context in further studies, the representation of primary, secondary and tertiary sectors should be evenly distributed.

In terms of methods, most of the studies included were qualitative in design, suggesting that this topic can be adequately explored using interviews or focus groups. Further quantitative studies are recommended to verify the subjective information provided by theses qualitative methods. Representative results on the needs of many MSMEs regarding occupational health services might have a greater impact on the optimisation and adaption of services by occupational health professionals.

### Strength and limitation

Occupational health services, especially in micro-, small- and medium-sized enterprises with unequal provision are of great importance in part due to the large number of employees affected by changing working conditions. To improve the provision, this scoping review presented an overview of MSMEs’ needs regarding occupational health services in a broader sense as summarised in the scientific literature. It could be emphasized that the identification of felt and expressed needs was based on direct assessment of MSMEs, as the included studies investigated the perspective of these enterprises. Another strength of this scoping review is the methodological compliance with the JBIs’ PRISMA checklist. Two researchers independently screened the abstract, title and full text and discussed the inconsistencies. This process is mandatory for systematic reviews, but not for scoping reviews. The research team invested time and effort to improve the quality of this scoping review by screening through a double check. This was also the reason for conducting a critical appraisal using the JBI tools. Moreover, the preregister with the Open Science Framework ensured transparency.

It should be noted that most of the included studies used a qualitative methodological approach. In the context of exploring felt and expressed needs of individuals, this approach can be appropriate, as it allows direct engagement with MSME managers and professionals to gain insight into their perspectives. Nevertheless, quantitative approaches may provide more generalizable data. Therefore, the combination of both approaches in this scoping review could be considered a strength.

Nevertheless, there are some limitations particularly in the methodological part. For instance, the search was restricted to four databases and was conducted only once, in German and English, due to limited resources. With additional time and resources, it would be advisable to extend the search to databases such as the Cochrane Library or PsycINFO and to include more languages to enable a more comprehensive approach. It should be mentioned that the search string was developed inductively by using different literature to collect the keywords and topic blocks, and therefore evident sources might be overlooked. Furthermore, the exclusion of grey literature represents a potential limitation, as relevant information may be overlooked. However, the decision to focus on peer-reviewed studies was made to ensure a high standard of methodological quality and reliability in the evidence presented.

Often, the included studies only indirectly addressed the needs of the MSMEs or had set other prioritised objectives. In most cases, the status quo of the occupational health, safety, prevention or psychosocial risk assessment was presented so that the author could interpret the needs from the results. Despite the eligibility criteria and the focus on essential bibliographic fields, there remains a risk of selection bias during the title-abstract and full-text screening. Although this process was conducted by two researchers, the possibility of selection bias and thus the omission of relevant articles, cannot be fully excluded.

The critical appraisal was conducted by a single reviewer only. Nevertheless, in the context of a scoping review, a critical appraisal is not necessarily required and is often considered an additional, optional step. Regarding the score, only two studies achieved more than 90%. Most studies met 70% of the methodological quality categories of the JBI tools, while the lowest score was 63%, which was considered sufficient quality in this scoping review. Due to the small number of included studies, all results were used in this scoping review, despite the lack of statements on the cultural location of the researchers and its influence on the qualitative studies or the absence of explanations of confounding factors in the quantitative studies. It should be mentioned that the JBI’ tools did not give any indication of what score is sufficient. Further studies should improve the quality of their methods by describing the influence and cultural location of the researchers or taking confounding factors into account.

In addition to these limitations, the data charting and synthesis process of this scoping review was performed by only one researcher due to resource limits. To maintain the content, the data charting process and synthesis results were discussed within the research team.

Delving deeper into the results, the categories were formed narratively and thus may be biased due to subjectivity and individuality of the first author. However, the categories and subcategories were also discussed within the research team. Nevertheless, the generalizability of the findings is limited, as most results pertain to the secondary sector within Europe. Consequently, the recommendations and conclusions should be interpreted within this specific context and in relation to the occupational health systems of the respective countries.

## Conclusion

To optimise the distribution of occupational health, safety and prevention in micro-, small- and medium-sized enterprises due to the impact of changing working environments on the health of many employees, this scoping review gives an overview of the needs of these enterprises. Based on the results of the 17 included studies with critical appraisal, five categories of felt and expressed needs were identified using narrative synthesis.

MSMEs need collaborative culture, knowledge and competencies, organizational structures, political and regulatory context adjustments regarding occupational health, safety and prevention. Practical recommendations for political and occupational professional stakeholders are to improve in these categories to optimise the implementation of MSMEs’ occupational health, safety and prevention. These findings cannot be generalised due to sectoral and legal differences.

However, this scoping review provides an overview in line with the JBI’s PRISMA checklist, maps the available information and shows that further research should be conducted with the explicit aim of identifying the needs of small enterprises in each sector on occupational health services, psychosocial risk and assessments.

## Supplementary Information

Additional file 1: PRISMA-ScR Checklist.

Additional file 2: Inclusion and exclusion criteria.

Additional file 3: Search Strategy for all databases.

Additional file 4: Excluded studies.

Additional file 5: Critical appraisal results.

## Data Availability

The included studies are listed within this article. Additional material is attached to the supplementary material. Further information is available from the corresponding author on request.

## Abbreviations

MSMEs: Micro-, small- and medium-sized enterprise
PCC: Population, Concept, Context framework
JBI: Joanna Briggs Institute
PRISMA-ScR: Preferred Reporting Items for Systematic reviews and Meta-Analyses extension for Scoping Reviews
PRESS: Peer Review of Electronic Search Strategies
