# Supplementary material for: Felt and expressed needs regarding occupational health services from the perspective of micro-, small and medium-sized enterprises: a scoping review
Source: BMC Public Health. 2026 Aug 5;26:2263. doi: 10.1186/s12889-026-28795-y (PMC13440008; doi:10.1186/s12889-026-28795-y)
Supplement: Supplementary file 2 [file 12889_2026_28795_MOESM2_ESM.pdf]

Additional file 2: Inclusion and exclusion criteria

	Inclusion criteria	Exclusion criteria
**Population**	Perspective of micro, small and medium-sized (1-250 employees)	• Perspective of the occupational health professionals<br>• Other stakeholders
**Concept**	Article about<br>• Occupational health services in MSMEs<br>○ Primary, secondary, tertiary prevention<br>○ Focus on the behaviour or the working environment<br>• Workplace environment/conditions<br>○ Factors influencing injuries<br>○ Risks and hazards to which personnel are exposed<br>• Health status/ experience/ satisfaction of the employees<br>• Challenges and goals of occupational health<br>• Effectiveness analysis of certain occupational health interventions<br><br>Focus on<br>needs of MSMEs with regard to occupational health services	No focus on<br>• Specific occupational illnesses/injuries/accidents<br>• Workplace health promotion<br>• Supporting personnel development<br>• Telemedicine/digital applications in occupational medicine<br>• General healthcare/primary medicine/primary care without reference to the workplace<br>• The situation during the Covid-19 pandemic<br><br>Justification: Omitted aspects are separate research areas
**Context**	Micro, small and medium-sized enterprises in all sectors (1-250 employees)<br>All geographical regions	Large companies/ enterprises (>250 employees)
**Period**	2000-2024	Before 2000
**Language**	English, German	Other languages
**Accessability**	Title, abstract and fulltext	No full text available
**Publication type**	Original article (qualitative/quantitative primary research)	Reviews, editorials, letters, conference papers, dissertation abstracts, commentaries, reflection, books, policy statements
**Publication status**	Peer-reviewed	No peer-review, unpublished articles
