# Supplementary material for: Felt and expressed needs regarding occupational health services from the perspective of micro-, small and medium-sized enterprises: a scoping review
Source: BMC Public Health. 2026 Aug 5;26:2263. doi: 10.1186/s12889-026-28795-y (PMC13440008; doi:10.1186/s12889-026-28795-y)
Supplement: Supplementary file 3 [file 12889_2026_28795_MOESM3_ESM.pdf]

Additional file 3: Search Strategy for all Databases

Database	Search string	Filter	Results	Research date
**Embase**	('occupational health':ti,ab OR 'occupational safety':ti,ab OR 'occupational medic*':ti,ab OR 'ohs':ti,ab OR 'osh':ti,ab OR 'ohsm':ti,ab OR 'ohp':ti,ab OR 'ohra':ti,ab OR 'workplace health':ti,ab OR 'workplace medicine':ti,ab OR 'industrial health':ti,ab) AND ('small':ti,ab OR 'micro':ti,ab OR 'medium':ti,ab OR 'sse':ti,ab OR 'mse':ti,ab OR 'msme':ti,ab OR 'sme':ti,ab) AND ('compan*':ti,ab OR 'business*':ti,ab OR 'enterprise*':ti,ab OR 'organisation*':ti,ab) AND ('needs':ti,ab OR 'wants':ti,ab OR 'requirements':ti,ab OR 'demands':ti,ab OR 'desire':ti,ab OR 'wish':ti,ab OR 'request':ti,ab OR 'longing':ti,ab OR 'dependence':ti,ab OR 'necessity':ti,ab OR 'condition':ti,ab OR 'basis':ti,ab)	2000-2024	191	26.08.2024
**PubMed**	("occupational health"[Title/Abstract] OR "occupational safety"[Title/Abstract] OR "occupational medic*"[Title/Abstract] OR "OHS"[Title/Abstract] OR "OSH"[Title/Abstract] OR "OHSM"[Title/Abstract] OR "OHP"[Title/Abstract] OR "OHRA"[Title/Abstract] OR "workplace health"[Title/Abstract] OR "workplace medicine"[Title/Abstract] OR "industrial health"[Title/Abstract]) AND ("small"[Title/Abstract] OR "micro"[Title/Abstract] OR "medium"[Title/Abstract] OR "SSE"[Title/Abstract] OR "MSE"[Title/Abstract] OR "MSME"[Title/Abstract] OR "SME"[Title/Abstract]) AND ("compan*"[Title/Abstract] OR "business*"[Title/Abstract] OR "enterprise*"[Title/Abstract] OR "organisation*"[Title/Abstract]) AND ("needs"[Title/Abstract] OR "wants"[Title/Abstract] OR "requirements"[Title/Abstract] OR "demands"[Title/Abstract] OR "desire"[Title/Abstract] OR "wish"[Title/Abstract] OR "request"[Title/Abstract] OR "longing"[Title/Abstract] OR "dependence"[Title/Abstract] OR "necessity"[Title/Abstract] OR "condition"[Title/Abstract] OR "basis"[Title/Abstract])	2000-2024	172	26.08.2024

Database	Search string	Filter	Results	Research date
**Scopus**	(TITLE-ABS("occupational health") OR TITLE-ABS("occupational safety") OR TITLE-ABS("occupational medic*") OR TITLE-ABS("OHS") OR TITLE-ABS("OSH") OR TITLE-ABS("OHSM") OR TITLE-ABS("OHP") OR TITLE-ABS("OHRA") OR TITLE-ABS("workplace health") OR TITLE-ABS("workplace medicine") OR TITLE-ABS("industrial health")) AND (TITLE-ABS("small") OR TITLE-ABS("micro") OR TITLE-ABS("medium") OR TITLE-ABS("SSE") OR TITLE-ABS("MSE") OR TITLE-ABS("MSME") OR TITLE-ABS("SME")) AND (TITLE-ABS("compan*") OR TITLE-ABS("business*") OR TITLE-ABS("enterprise*") OR TITLE-ABS("organisation*")) AND (TITLE-ABS("needs") OR TITLE-ABS("wants") OR TITLE-ABS("requirements") OR TITLE-ABS("demands") OR TITLE-ABS("desire") OR TITLE-ABS("wish") OR TITLE-ABS("request") OR TITLE-ABS("longing") OR TITLE-ABS("dependence") OR TITLE-ABS("necessity") OR TITLE-ABS("condition") OR TITLE-ABS("basis"))	2000-2024	669	26.08.2024
**Web of Science**	((TI=("occupational health") OR TI=("occupational safety") OR TI=("occupational medic*") OR TI=("OHS") OR TI=("OSH") OR TI=("OHSM") OR TI=("OHP") OR TI=("OHRA") OR TI=("workplace health") OR TI=("workplace medicine") OR TI=("industrial health")) OR (AB=("occupational health") OR AB=("occupational safety") OR AB=("occupational medic*") OR AB=("OHS") OR AB=("OSH") OR AB=("OHSM") OR AB=("OHP") OR AB=("OHRA") OR AB=("workplace health") OR AB=("workplace medicine") OR AB=("industrial health"))) AND ((TI=("small") OR TI=("micro") OR TI=("medium") OR TI=("SSE") OR TI=("MSE") OR TI=("MSME") OR TI=("SME")) OR (AB=("small") OR AB=("micro") OR AB=("medium") OR AB=("SSE") OR AB=("MSE") OR AB=("MSME") OR AB=("SME"))) AND ((TI=("compan*") OR TI=("business*") OR TI=("enterprise*") OR TI=("organisation*")) OR (AB=("compan*") OR AB=("business*") OR AB=("enterprise*") OR AB=("organisation*"))) AND ((TI=("needs") OR TI=("wants") OR TI=("requirements") OR TI=("demands") OR TI=("desire") OR TI ("wish") OR TI=("request") OR TI=("longing") OR TI=("dependence") OR TI=("necessity") OR TI=("condition") OR TI=("basis")) OR (AB=("needs") OR AB ("wants") OR AB=("requirements") OR AB=("demands") OR AB=("desire") OR AB=("wish") OR AB=("request") OR AB=("longing") OR AB=("dependence") OR AB=("necessity") OR AB=("condition") OR AB=("basis")))	2000-2024	220	26.08.2024
**Results**			**1.252**	
