# Supplementary material for: Felt and expressed needs regarding occupational health services from the perspective of micro-, small and medium-sized enterprises: a scoping review
Source: BMC Public Health. 2026 Aug 5;26:2263. doi: 10.1186/s12889-026-28795-y (PMC13440008; doi:10.1186/s12889-026-28795-y)
Supplement: Supplementary file 4 [file 12889_2026_28795_MOESM4_ESM.pdf]

Additional file 4: Excluded studies

Database search

Language other than German or English (*n* = 3),

Assunção, A. A. and Sampaio, R. F. and Nascimento, L. M. B. Actions in small companies to promote occupational health: The case of the food and beverage sector Revista Brasileira de Fisioterapia 2010

Fasikov, R. M. and Stepanov, E. G. Importance of occupational medicine problem on small and medium scale business enterprises Meditsina truda i promyshlennaia ekologiia 2008

Mosconi, G. Health surveillance in the handicraft and small enterprises: Which organisation? Giornale Italiano di Medicina del Lavoro ed Ergonomia 2009

Publication type (*n* = 7)

Bogna, F. and Dell, G. and Raineri, A. Incorporating internal context into the design of occupational health and safety research and intervention programmes in SMEs Small Enterprise Research 2018 **(Review)**

Cagno, E. and Micheli, G. J. L. and Perotti, S. Identification of OHS-related factors and interactions among those and OHS performance in SMEs Safety Science 2011

Eakin, J. M. and Champoux, D. and MacEachen, E. Health and safety in small workplaces: refocusing upstream Canadian journal of public health. Revue canadienne de santé publique 2010

Floyde, A. and Lawson, G. and Shalloe, S. and Eastgate, R. and D'Cruz, M. The design and implementation of knowledge management systems and e-learning for improved occupational health and safety in small to medium sized enterprises Safety Science 2013

Lourenço, M. and Lima, T. M. and Gaspar, P. D. and Santos, F. C. Assessment and improvement opportunities for occupational health and safety in the Portuguese food processing industry Studies in Systems, Decision and Control 2019

Mayhew, C. OHS in Australian "micro" small businesses: Evidence from nine research studies Journal of Occupational Health and Safety - Australia and New Zealand 2000

Santos, G. and Rebelo, M. and Barros, S. and Silva, R. and Pereira, M. and Ramos, G. and Lopes, N. Developments regarding the integration of the occupational safety and health with quality and environment management systems Occupational Safety and Health 2014

Intervention design (*n* = 7)

Bejan, A. and Parker, D. L. and Brosseau, L. M. and Xi, M. and Skan, M. A comparison of owner and expert evaluation of health and safety in small collision repair shops: A pilot study International Journal of Occupational and Environmental Health 2013

Hansen, E. and Landstad, B. J. and Gundersen, K. T. and Vinberg, S. Leader-Based Workplace Health Interventions - A Before-After Study in Norwegian and Swedish Small-Scale Enterprises International Journal of Disability Management 2016

Huỳnh, T. B. and Nguyễn Nguyá, D. and Vũ, N. and Carroll-Scott, A. and Wong, C. and Freeland, C. and Parvanta, C. Perceived Benefits and Barriers to Implementing Occupational Health Recommendations Among Immigrant-Owned Nail Salons in the Greater Philadelphia Region Health Promotion Practice 2024

Limborg, H. and Grøn, S. and Flensborg Jensen, M. Research Note: Networking among small and medium-sized enterprises: Meeting the challenge of promoting safety and health measures Small Enterprise Research 2014

Martin, A. and Sanderson, K. and Scott, J. and Brough, P. Promoting mental health in small-medium enterprises: an evaluation of the "Business in Mind" program BMC Public Health 2009

Valsecchi, R. and Anderson, N. and Balta, M. E. and Harrison, J. Managing Health and Well-Being in SMEs through an Adviceline: A Typology of Managerial Behaviours Work, Employment and Society 2023

Yalala, N. N. The state of OSH in Small Scale Enterprises of Bulawayo: The case of OK Mart SMEs Safety and Health at Work 2022

Perspective of occupational health professionals and not the perspective of the MSMEs (*n* = 9)

Bruening, R. A. and Strazza, K. and Nocera, M. and Peek-Asa, C. and Casteel, C. Understanding small business engagement in workplace violence prevention programs Am J Health Promot 2015

Hagqvist, E. and Vinberg, S. and Toivanen, S. and Hagstrom, M. and Granqvist, S. and Landstad, B. J. Falling outside the system: Occupational safety and health inspectors’ experiences of micro-enterprises in Sweden Safety Science 2020

Hogg, B. and Moreno-Alcázar, A. and Tóth, M. D. and Serbanescu, I. and Aust, B. and Leduc, C. and Paterson, C. and Tsantilla, F. and Abdulla, K. and Cerga-Pashoja, A. and Cresswell-Smith, J. and Fanaj, N. and Meksi, A. and Ni Dhalaigh, D. and Reich, H. and Ross, V. and Sanches, S. and Thomson, K. and Van Audenhove, C. and Pérez, V. and Arensman, E. and Purebl, G. and Amann, B. L. and Connor, A. and Szekely, A. and LaMontagne, A. and Como, A. and Dushaj, A. and Doukani, A. and Justicia, A. and Greiner, B. A. and Lockwood, C. and Connor, C. and McDaid, D. and Kim, D. and Williamson, E. and Griffin, E. and Coppens, E. and Burazeri, G. and Qirjako, G. and Davey, G. and van Weeghel, J. and Eustace, J. and Kilroy, J. and Hug, J. and Kolves, K. and Mulcahy, K. and Michell, K. and Wahlbeck, K. and de Winter, L. and Cox, L. and Alessandro, L. and Maxwell, M. and Reavley, N. and Trembeczky, P. and Corcoran, P. and Rugulies, R. and Benson, R. and Rapeli, S. and Ihinonvien, S. and Mustafa, S. and Mathieu, S. and Hackel, S. and King, T. and Hegerl, U. and Scott, V. and Orchard, W. Supporting employees with mental illness and reducing mental illness-related stigma in the workplace: an expert survey European Archives of Psychiatry and Clinical Neuroscience 2023

Izumi, M. Occupational health service and activities for workers of small-scale enterprises comparative study of South Korea and Japan Occupational and Environmental Medicine 2018

Jaroenroy, T. and Chompunth, C. An alternative integrated occupational health, safety and environmental management system for small and medium-sized enterprises (SMEs) in Thailand International Journal of GEOMATE 2019

Moriguchi, J. and Ikeda, M. and Sakuragi, S. and Takeda, K. and Muto, T. and Higashi, T. and Weel, A. N. H. and Van Dijk, F. J. Activities of occupational physicians for occupational health services in small-scale enterprises in Japan and in the Netherlands International Archives of Occupational and Environmental Health 2010

Syahri, I. M. and Utama, S. Stakeholder collaboration in improving integrated basic occupational in health services supply chain in community health center Uncertain Supply Chain Management 2021

Vinberg, S. and Torsdatter Markussen, L. and Landstad, B. J. Cooperation Between Occupational Health Services and Small-Scale Enterprises in Norway and Sweden: A Provider Perspective Workplace Health Saf 2017

Wang, Y. H. and Chang, C. M. and Liao, H. C. Reducing waste in healthcare through occupational safety and health measures: A study of manufacturing industries in taiwan Healthcare (Switzerland) 2021

Status quo without mentioning the needs of MSMEs (*n* = 8)

Campanelli, L. C. and Ribeiro, L. D. and Campanelli, L. C. Involvement of Brazilian companies with occupational health and safety aspects and the new ISO 45001:2018 Production 2021

Champoux, D. and Brun, J. P. OSH practices and interventions in small businesses: Global issues in the Quebec contextPolicy and Practice in Health and Safety 2015

Dugolli, M. Occupational, health and safety situation at small and medium enterprises in Kosovo, contextual factors, barriers, drivers and intervention process International Review of Applied Sciences and Engineering 2021

Esterhuyzen, E. Occupational health and safety in small businesses: The rationale behind compliance AFRICAN JOURNAL OF BUSINESS ETHICS 2022

Landstad, B. J. and Vinberg, S. and Rahme, A. and Vigren, G. and Hagqvist, E. Management by values: A qualitative study of how small business owners in the cleaning sector view and implement their employer responsibilities with respect to occupational safety and health management Safety Science 2022

Randrianarivelo, F. and Badri, A. and Gauthier, F. and Boudreau-Trudel, B. Prevention of Occupational Diseases in Small and Medium-Sized Manufacturing Enterprises in QUEBEC (CANADA) - Study of Prevention Management Practices International Journal of Safety and Security Engineering 2022

Schmidt, L. and Gunnarsson, K. and Dellve, L. and Antonsson, A. B. Utilizing occupational health services in small-scale enterprises: a 10-year perspective Small Enterprise Research 2016

Vu, G. K. and Le, T. A. and Ngoc, T. P. and Nguyen, T. H. T. and Ha-Nguyen, M. Q. Risk assessment, implementation of occupational health, safety and hygiene in small and medium manufacturing enterprises: A case study in central Vietnam Decision Science Letters 2023

Workplace health promotion (*n* = 7)

Binder, L. and Coenen, M. and von Mallek, P. and Kus, S. Creating a healthy work environment: what do employees in small and medium-sized enterprises need in rural areas? Presenting a cross-industry perspective using a mixed method approach Pravention und Gesundheitsforderung 2024

Hughes, M. C. and Patrick, D. L. and Hannon, P. A. and Harris, J. R. and Ghosh, D. L. Understanding the decision-making process for health promotion programming at small to midsized businesses Health Promot Pract 2011

Lutz, R. and Fischmann, W. and Drexler, H. and Nehammer, E. A German Model Project for Workplace Health Promotion Flow of Communication, Information, and Reasons for Non-Participation in the Offered Measures International Journal of Environmental Research and Public Health 2022

Schaefer, E. and Drexler, H. and Kiesel, J. Workplace Health Promotion in Small, Medium-Sized and Large Enterprises of the Health-Care Sector - Frequency, Reasons for the Company Management to Take Action and Barriers to Implementation Gesundheitswesen 2016

Vinberg, S. and Hansen, E. and Hedlund, M. and Landstad, B. J. Ambiguity among managers in small-scale enterprises: How to handle business and workplace health management Societies 2017

Landstad, B. J. and Hedlund, M. and Vinberg, S. How managers of small-scale enterprises can create a health promoting corporate culture International Journal of Workplace Health Management 2017

Wiman, V. and Lydell, M. and Nyholm, M. Views of the workplace as a health promotion arena among managers of small companies Health Education Journal 2016

Topics not relevant (*n* = 13)

Irvine, A. and Suter, J. Managing mental health problems in the workplace: are small businesses different? Employee Relations 2023

Legg, S. and Olsen, K. and Lamm, F. and Laird, I. and Harris, L. A. and Hasle, P. Understanding the programme theories underlying national strategies to improve the working environment in small businesses POLICY AND PRACTICE IN HEALTH AND SAFETY 2010

Lentz, T. J. and Sieber, W. K. and Jones, J. H. and Piacitelli, G. M. and Catlett, L. R. Occupational health and safety surveillance: Surveillance of safety and health programs and needs in small U.S. businesses Applied Occupational and Environmental Hygiene 2001

Makri, E. and Walters, D. and Wadsworth, E. and Devereux, H. and Van Den Burg, S. W. K. Occupational health and safety in aquaculture: Organisation of work and employment in small seaweed farms in North West Europe Economic and Labour Relations Review 2024

Md Deros, B. and Rasdan Ismail, A. and Ghani, J. A. and Mohd Yusof, M. Y. Conformity to occupational safety and health regulations in Malaysian small and medium enterprises American Journal of Applied Sciences 2014

Oluranti, S. Perceptions of occupational health and safety in small and medium scale enterprises in Lagos State, and the Nigerian OHS laws Occupational and Environmental Medicine 2018

Petković, A. I. and Nikolić, V. Educational needs of employees in work-related stress management Work 2020

Schuller, K. It's good that we talked about it ...?! Methodological challenges in the assessment of risk of psychological stress in SMEs Arbeitsmedizin Sozialmedizin Umweltmedizin 2018

Tint, P. and Paas, O. and Reinhold, K. Cost-effectiveness of safety measures in enterprises Engineering Economics 2010

Vickers, I. and James, P. and Smallbone, D. and Baldock, R. Understanding small firm responses to regulation: The case of workplace health and safety Policy Studies 2005

Wagner, A. and Tsarouha, E. and Ã–g, E. and Preiser, C. and Rieger, M. A. and Rind, E. Work-related psychosocial demands related to work organization in small sized companies (SMEs) providing health-oriented services in Germany a qualitative analysis BMC Public Health 2022

Winkler, E. Job demands and resources of managing directors of micro- and small enterprises: A qualitative study of the German transport industry Work (Reading, Mass.) 2022

Zulkkifly, S. S. and Baharudin, M. R. and Mahadi, M. R. and Hasan, N. H. and Ismail, S. N. S. The Impact of Superior Roles in Safety Management on Safety Performance in SME Manufacturing in Malaysia Global Business Review 2021

Manual search

Publication type (*n* = 9)

Antonnson L, Birgersdotter AB, Bornberger-Dankvardt S. Small enterprises in Sweden – health and safety and the significance of intermediaries in preventive health and safety. Stockholm: National Institute for Working Life; 2002.

Cacace M, Franz I, Braun-Beustrin D, Ratz D. Smalland medium-sized enterprises' preferences for occupational health services and willingness to pay. In: Wiencke M, Carace M, Fischer S, editors. Healthy at Work. Cham: Springer; 2016. pp. 197-215.

Eakin JM, Lamm P, Limborg HJ. 2000. International perspectives on the promotion of health and safety in small workplaces. In: Frick K. JPL, Quinlan M, Wilthagen T, editors. Systematic OHS management: Perspectives on an international development. Amsterdam, The Netherlands: Elsevier. p 227–247

Glass W. Small enterprises and occupational health and safety: development, technology and trade. In: Stellman JM, editor. Encyclopaedia of occupational health and safety. Vol. 20. Geneva: International Labour Office; 2011. p. 20.8–20.9.

Hasle, P., & Limborg, H. J. (2006). A review of the literature on preventive occupational health and safety activities in small enterprises. Industrial Health, 44(1), 6-12.

Rantanen J, Warshaw LJ. Occupational health services in small-scale enterprises. In: Stellman JM, editor. Encyclopaedia of occupational health and safety. Vol. 16. Geneva: International Labour Office; 2011. p.16.25–16.27.

Sczesny C, Keindorf S, Droß P, Jasper G: Kenntnisstand von Unternehmen und Beschäftigten auf dem Gebiet des Arbeits- und Gesundheitsschutzes in KMU. Dortmund/Berlin/Dresden, 2014

Sczesny C, Keindorf S, Droß P: Kenntnisstand von Unternehmen auf dem Gebiet des Arbeits- und Gesundheitsschutzes in KMU – Ergebnisse einer repräsentativen Befragung von Inhaber/innen / Geschäftsführer/innen in Klein- und Kleinstunternehmen. 1. Aufl. Dortmund: Bundesanstalt für Arbeitsschutz und Arbeitsmedizin, 2011.

Walters, D.; Wadsworth, E.; Hasle, P.; Refslund, B.; Ramioul, M.; Antonsson, A.-B. Safety and Health in Micro and Small Enterprises in the EU: The View from the Workplace; European Agency for Safety and Health at Work (OSHA): Luxembourg, 2018.

Perspective of occupational health professionals and not the perspective of the MSMEs (*n* = 2)

Cagno E, Micheli GJL, Perotti S. Identification of OHSrelated factors and interactions among those and OHS performance in SMEs. Saf Sci. 2011; 49:216–225.

Hasle, P.; Refslund, B.; Antonsson, A.; Ramioul, M.; Walters, D. Safety and Health in Micro and Small Enterprises in the EU: From Policy to Practice; European Agency for Safety and Health at Work (OSHA) Publications Office: Luxembourg, 2017

Intervention design (*n* = 3)

Arocena P, Núñez I. An empirical analysis of the effectiveness of occupational health and safety management systems in SMEs. Int Small Bus J. 2010;28(4):398–419.

Dawkins S, Martin A, Kilpatrick M, Scott J. Reasons for engagement: SME owner-manager motivations for engaging in a workplace mental health and wellbeing intervention. J

Occup Environ Med. 2018those and OHS performance in SMEs. Saf Sci. 2011; 49:216–225.

Walker D, Tait R. Health and safety management in small enterprises: an effective low-cost approach. Saf Sci. 2004; 42:69–83.

Status quo without mentioning the needs of MSMEs (*n* = 3)

Hasle P. Health and safety in small enterprises in Denmark and the role of intermediaries. Copenhagen: Centre for Alternative Social Analysis; 2000.

Hasle, P., Limborg, H.J., Kallehave, T., Klitgaard, C., Andersen, T.R., 2012a. The working environment in small firms: Responses from owner-managers. Int. Small Bus. Journal 30, 622–639.

O'Hara R, Dickety N, Weyman A. Good practice in assessing workplace risks by small and medium-sized enterprises. Risk Manag. 2005;7(1):31–41
