# Supplementary material for: Felt and expressed needs regarding occupational health services from the perspective of micro-, small and medium-sized enterprises: a scoping review
Source: BMC Public Health. 2026 Aug 5;26:2263. doi: 10.1186/s12889-026-28795-y (PMC13440008; doi:10.1186/s12889-026-28795-y)
Supplement: Supplementary file 5 [file 12889_2026_28795_MOESM5_ESM.pdf]

Additional file 5: Critical appraisal results

Table 1 Critical appraisal results of qualitative studies

**Qualitative categories**<br>✓ = criterion met<br>X = criterion not met<br>(?) = criterion unclear	**Amler et al. (2019)**	**Barbeau et al. (2004)**	**Benning et al. (2022)**	**Cagno et al. (2016)**	**Champoux and Brun (2003)**	**Holt and Powell (2015)**	**Kvorning et al. (2015)**	**Masi and Cagno (2015)**	**Masi et al. (2014)**	**Micheli and Cagno (2008)**	**Micheli et al. (2018)**	**Pavlista et al. (2018)**	**Stynen et al. (2023)**	**Zimber et al. (2024)**
1. Is there congruity between the stated philosophical perspective and the research methodology?	(?)	(?)	(?)	(?)	(?)	(?)	✓	✓	(?)	(?)	(?)	(?)	(?)	(?)
2. Is there congruity between the research methodology and the research question or objectives?	✓	✓	✓	✓	✓	✓	✓	✓	✓	✓	✓	✓	✓	✓
3. Is there congruity between the research methodology and the methods used to collect data?	✓	✓	✓	✓	✓	✓	✓	✓	✓	✓	✓	✓	✓	✓
4. Is there congruity between the research methodology and the representation and analysis of data?	✓	✓	✓	✓	✓	✓	✓	✓	✓	✓	✓	✓	✓	✓

**Qualitative categories**<br>✓ = criterion met<br>X = criterion not met<br>(?) = criterion unclear	**Amler et al. (2019)**	**Barbeau et al. (2004)**	**Benning et al. (2022)**	**Cagno et al. (2016)**	**Champoux and Brun (2003)**	**Holt and Powell (2015)**	**Kvorning et al. (2015)**	**Masi and Cagno (2015)**	**Masi et al. (2014)**	**Micheli and Cagno (2008)**	**Micheli et al. (2018)**	**Pavlista et al. (2018)**	**Stynen et al. (2023)**	**Zimber et al. (2024)**
5. Is there congruity between the research methodology and the interpretation of results?	✓	✓	✓	✓	✓	✓	✓	✓	✓	✓	✓	✓	✓	✓
6. Is there a statement locating the researcher culturally or theoretically?	X	X	X	X	X	X	X	X	X	X	X	X	X	✓
7. Is the influence of the researcher on the research, and vice-versa, addressed?	X	X	X	X	X	X	X	X	X	X	X	✓	X	✓
8. Are participants, and their voices, adequately represented?	✓	✓	✓	✓	✓	✓	✓	✓	✓	✓	✓	✓	✓	✓
9. Is the research ethical according to current criteria or, for recent studies, and is there evidence of ethical approval by an appropriate body?	✓	✓	✓	✓	✓	✓	✓	✓	✓	✓	✓	✓	✓	✓

**Qualitative categories**<br>✓ = criterion met<br>X = criterion not met<br>(?) = criterion unclear	**Amler et al. (2019)**	**Barbeau et al. (2004)**	**Benning et al. (2022)**	**Cagno et al. (2016)**	**Champoux and Brun (2003)**	**Holt and Powell (2015)**	**Kvorning et al. (2015)**	**Masi and Cagno (2015)**	**Masi et al. (2014)**	**Micheli and Cagno (2008)**	**Micheli et al. (2018)**	**Pavlista et al. (2018)**	**Stynen et al. (2023)**	**Zimber et al. (2024)**
10. Do the conclusions drawn in the research report flow from the analysis, or interpretation, of the data?	✓	✓	✓	✓	✓	✓	✓	✓	✓	✓	✓	✓	✓	✓
**Critical appraisal percentage**	**70%**	**70%**	**70%**	**70%**	**70%**	**70%**	**80%**	**70%**	**80%**	**70%**	**70%**	**80%**	**70%**	**90%**

Table 2 Critical appraisal results of quantitative studies

**Quantitative categories**<br>✓ = criterion met<br>X = criterion not met	**Amler et al. (2019)**	**Benning et al. (2022)**	**Coppens et al. (2023)**	**Garnica and Barriga (2018)**	**Micheli et al. (2018)**	**Nowrouzi et al. (2016)**
1. Were the criteria for inclusion in the sample clearly defined?	✓	✓	✓	✓	✓	✓
2. Were the study subjects and the setting described in detail?	✓	✓	✓	✓	X	✓
3. Was the exposure measured in a valid and reliable way?	✓	✓	✓	✓	✓	✓
4. Were objective, standard criteria used for measurement of the condition?	✓	✓	✓	✓	✓	✓
5. Were confounding factors identified?	X	X	X	X	X	✓
6. Were strategies to deal with confounding factors stated?	X	X	X	X	X	✓
7. Were the outcomes measured in a valid and reliable way?	✓	✓	✓	✓	✓	✓
8. Was appropriate statistical analysis used?	✓	✓	✓	✓	✓	✓
**Critical appraisal percentage**	**75%**	**75%**	**75%**	**75%**	**63%**	**100%**
